# Attributions of Responsibility and Blame for Procrastination Behavior

**DOI:** 10.3389/fpsyg.2016.01179

**Published:** 2016-08-05

**Authors:** Sonia Rahimi, Nathan C. Hall, Timothy A. Pychyl

**Affiliations:** ^1^Department of Educational and Counselling Psychology, McGill University, Montreal, QCCanada; ^2^Department of Psychology, Carleton University, Ottawa, ONCanada

**Keywords:** academic procrastination, moral responsibility, blame, attribution theory, experimental philosophy

## Abstract

The present study examined the relationship between procrastination, delay, blameworthiness, and moral responsibility. Undergraduate students (*N* = 240) were provided two scenarios in which the reason for inaction (procrastination, delay), the target (self, other), and the outcome (positive, negative) were manipulated, and students were asked to rate the moral responsibility and blameworthiness of the agent. Results indicated that individuals who procrastinated were seen as more morally responsible and blameworthy than those who experienced delay. More specifically, after a negative outcome, procrastination was associated with more moral responsibility, whereas delay was associated with less moral responsibility. After a positive outcome, individuals perceived procrastination as deserving of less moral responsibility, and delays as associated with more moral responsibility. Finally, a three-way interaction showed that participants rated procrastination that resulted in failure as deserving of responsibility when engaged in by others as opposed to oneself.

## Introduction

Procrastination is an everyday occurrence observed in various domains, and is particularly apparent in academic settings with an estimated 80–95% of college students reporting that they engage in this self-defeating behavior (Ellis and Knaus, 1977; Schouwenburg, 1995; O’Brien, unpublished doctoral dissertation) and an estimated 90% engaging in procrastination at least 1 hour a day (Klassen et al., 2008). Procrastination refers to the voluntary, needless delay of an intended act despite expecting negative consequences for this delay, and has been found to be strongly associated with emotions, such as guilt (Pychyl et al., 2000) and shame (Fee and Tangney, 2000; Wohl et al., 2010). These powerful negative emotions reflect a judgment about oneself with respect to moral responsibility, and are specific to procrastination as opposed to other forms of delay. While all procrastination is delay, not all delay is procrastination (Pychyl, 2013). In contrast to reasoned delay aimed at facilitating goal attainment in achievement settings, or external delays beyond one’s personal control, procrastination represents a needless gap between intention and action that is indicative of self-regulation failure (Sirois and Pychyl, 2013).

From an experimental philosophy perspective, findings indicate that individuals are more likely to perceive others as responsible for morally objectionable behaviors (seen as intentional) when they have negative consequences that lead to feelings of blame and punishment (Knobe, 2003). Similarly, social-psychological research based on attribution theory suggests that perceiving others as responsible for their negative experiences (seen as personally controllable) leads to more anger and less assistance (Weiner, 2006). Given the relevance of both philosophical and social-psychological research on perceived responsibility concerning blameworthy actions, this study evaluated students’ perceptions of responsibility and blameworthiness with respect to procrastination in educational settings as compared to experienced delay. Furthermore, the present study explored the extent to which students’ perceptions surrounding procrastination and delay were affected by the consequences of these behaviors (positive versus negative), as well as who experienced them (self versus others).

## Theoretical Background

### Procrastination Behavior

Procrastination is defined in psychological research as voluntarily delaying an intended course of action, despite the expectation of being worse off for that delay (Steel, 2007). Procrastination is also associated with attending to one’s immediate needs despite possible negative consequences (Sirois and Pychyl, 2013), potentially for motivational or affective reasons (Baumeister and Heatherton, 1996; Tice and Bratslavsky, 2000). Procrastination research further suggests that individuals are more likely to postpone tasks that are unpleasant (Milgram et al., 1988; Blunt and Pychyl, 2000) or when task rewards are more distal (Steel, 2007). Procrastination is thus commonly understood as occurring when individuals *cognitively* focus on their present self as opposed to their future self, and in so doing, sabotage their long-term emotional well-being and success by shifting the behavioral and psychological burden to their future self (Tice and Bratslavsky, 2000; Sirois and Pychyl, 2013; Krause and Freund, 2014). Moreover, procrastination is typically evaluated in relation to *affect*-related factors, with people being more likely to engage in avoidance behaviors as a deadline approaches (Wohl et al., 2010) to minimize negative emotions such as shame, guilt, and regret (Higgins et al., 1994; Fee and Tangney, 2000; Blunt and Pychyl, 2005).

This primacy of present self over future self, the tendency to “give in to feel good,” is characterized more broadly in psychological research as self-regulation failure (Baumeister et al., 1994; Tice and Bratslavsky, 2000). Self-regulation failure occurs despite goal-setting and self-monitoring due to an inordinate focus on escaping negative moods (i.e., Baumeister and Heatherton, 1996). Consequently, individuals often choose to repair their mood through avoidance behaviors such as procrastination (Sirois and Pychyl, 2013) as opposed to goal-oriented approach behaviors (Higgins et al., 1994). Procrastination has long been described in philosophical literature as a weakness of will, or “akrasia,” in which one acts against one’s better judgment (Searle, 2001; Kalis et al., 2008; Pychyl, 2011). Accordingly, self-regulation failure or akrasia can be explained as a conflict between what one feels they should be doing (duty) and what one is actually doing (action). Furthermore, this postponement of effort is understood as representing a lack of moral character – a direct violation of one’s responsibilities to oneself and others (Andreou and White, 2010) – underscoring the morally unacceptable nature of procrastination.

In educational settings, students who procrastinate tend to demonstrate greater negative affect (e.g., guilt, shame; Fee and Tangney, 2000), poorer study habits (e.g., Solomon and Rothblum, 1984), and lower grades relative to non-procrastinators (e.g., Klassen et al., 2008; Corkin et al., 2011; Kim and Seo, 2015). Academic procrastination is also typically viewed as resulting from low motivation (Senécal et al., 1995; Cerino, 2014), perfectionism (Burnam et al., 2014), and occurs when students do not enjoy academic tasks and perceive them to have little benefit (Schraw et al., 2007) or personal value (Fischer, 2001). As such, academic procrastination is commonly associated with detrimental behavioral choices as well as emotions implicating personal culpability, warranting further investigation of moral perceptions of this maladaptive strategy.

### An Experimental Philosophy Perspective

Research conducted in the experimental philosophy domain explores the nature of folk intuitions regarding traditional philosophical constructs, such as morality and free will, through the use of controlled experiments and statistical analyses (e.g., Knobe, 2006). Thus, whereas psychology research has focussed primarily on identifying the antecedents, correlates, and consequences of learners’ behaviors in academic settings, experimental philosophy researchers use similar techniques to evaluate individual’s general beliefs surrounding the intentionality and morality of these behaviors in themselves and others. More specifically, traditional philosophical perspectives suggest that an agent is viewed as morally responsible for an outcome if it results from an active choice – an exercise of free will (Kant, 1785). However, other philosophical work proposes that an agent may be deemed morally responsible for an outcome if that outcome is simply attributed to the agent as having caused it, regardless of volition or intent (Levy, 2005).

Recent work by experimental philosopher Joshua Knobe has addressed the stereotyped beliefs held by individuals concerning the intentionality, morality, and blameworthiness of the outcomes they experience (positive versus negative). In experimental research involving scenarios that examined the folk-psychological concept of intention, findings showed individuals’ beliefs concerning the intentionality of an outcome to be dependent on evaluative assumptions concerning the agent (Knobe, 2006). In these studies, participants were more likely to believe that an agent intentionally caused a morally reprehensible outcome (i.e., harming others) if the agent knew it might occur. Although the negative outcome may have not been explicitly intended (i.e., was a side-effect of an action having other benefits), it was anticipated by the agent as a possibility thus causing that negative outcome to be viewed as intentional by participants (Knobe, 2003). Conversely, participants did not view the side-effects of an action to be intentional if that outcome was morally respectable in nature (i.e., helping others). Overall, this research suggests that perceptions of intentionality for others’ behaviors tend to be biased by the moral significance of the outcome, even if the intentionality of the behavior is debatable.

## Judgments of Responsibility: Attribution Theory

Research from a social-psychological perspective has also addressed the extent to which individuals are judged as personally responsible for a given outcome, and more specifically, how stereotyped beliefs concerning intentionality can bias one’s explanations for why an event occurred. According to Weiner’s (2006) attribution theory, individuals’ perceptions of personal controllability over an outcome should predict judgments of responsibility that lead to specific emotions and behaviors. More specifically, if an event that happens to oneself is seen as personally controllable (e.g., lack of effort), one would typically believe they were responsible for the event, experience hope and guilt, and be more likely to persist in the future. In contrast, if one views an event experienced by another individual as controllable by that individual, one would likely perceive that individual as responsible, feel anger toward the person, and behave negatively toward them (e.g., neglect, reprimand, retaliation; Weiner, 2000).

However, research in social psychology has long demonstrated that the type of attribution individuals select may be biased by whether the event happened to oneself or another. As highlighted in classic research on the actor/observer bias (Jones and Nisbett, 1971), the hedonic bias, and the fundamental attribution error (Ross, 1977), individuals tend to attribute positive experiences to internal factors (within themselves) and negative experiences to external factors (outside themselves). For example, whereas a student would be expected to take credit for a good grade, they would further be expected to attribute a bad grade to test difficulty. Conversely, people tend to attribute negative events that happen to others primarily to internal factors (e.g., poor test performance of a peer being attributed to factors within that individual). Taken together, both social-psychological and experimental-philosophy perspectives suggest that people are more motivated to blame others for negative as opposed to positive outcomes, particularly when a behavior that contributed to the outcome is considered intentional in nature.

The social-psychological and experimental philosophy literatures are also similar in their shared emphasis on differentiating between cognitive and affective constructs when describing how people think about intentionality. In Weiner’s (2006) attribution theory, one’s cognitions surrounding the intentionality of an event (e.g., perceived controllability, responsibility) are presented as conceptually distinct from the emotions that follow (e.g., guilt, anger, sympathy). Similarly, perceptions of intentionality or responsibility are described by Knobe (2003) as leading to feelings of “blameworthiness,” a construct also described by Weiner (2006) as distinct from “affectively neutral” responsibility beliefs in conveying “emotional negativity” (due to its moral basis). Thus, although research in experimental philosophy to date does not distinguish between perceptions of intentionality for events that happen to oneself versus others (e.g., Knobe, 2003), findings from both philosophy and social psychology (e.g., Weiner, 2006) highlight how the moral relevance of a behavior or outcome can bias perceptions of its intentionality, and the importance of evaluating both cognitive and affective consequences of individuals’ beliefs concerning intentional behaviors (e.g., responsibility versus blame).

## The Present Study

The present study aimed to experimentally investigate students’ perceptions of responsibility and blameworthiness to address a current lack of research on how procrastination and its outcomes are perceived by oneself and others in educational settings. The scenario study protocols are consistent with relevant research in social psychology (Weiner et al., 1997) and experimental philosophy (Knobe, 2003, 2006) in evaluating students’ perceptions of intentionality with respect to (a) procrastination versus delays resulting in (b) a positive or negative outcome that (c) occurs to oneself or another. It was hypothesized that higher levels of perceived responsibly and blameworthiness would be observed for scenarios depicting (i) procrastination versus delay (Knobe, 2003), (ii) negative versus positive outcomes (Knobe, 2006; Weiner, 2006), and for (iii) others versus oneself (Ross, 1977). In addition, two-way interactions were anticipated with higher levels of perceived responsibility and blameworthiness expected for (iv) procrastination resulting in negative versus positive outcomes (Knobe, 2006; Weiner, 2006), and (v) for negative events that happen to others versus oneself (e.g., Ross, 1977; Weiner, 2006).

## Materials and Methods

The study sample was comprised of undergraduates (*N* = 240) recruited from first- and second-year psychology courses at a research-intensive Canadian university for an online study in exchange for course credit. Participants’ ages ranged from 17–32 years (*M*_age_ = 20), and the majority of participants were female (75%). After completing a web-based consent form, students were randomly presented a link to one of eight experimental conditions (2 × 2 × 2 study design), each requiring them to read two scenarios reflecting one combination of three factors outlined in the study hypotheses, namely (i) a behavior involving procrastination versus delays (e.g., due to external factors), (ii) a positive versus negative outcome of that behavior, and (iii) the situation involving oneself versus another individual. The specific scenario topics were primarily academic in nature (e.g., applying for student loans, applying for a research assistant position, conducting an SPSS analysis, renewing a driver’s license). Below is a sample scenario reflecting a procrastination event with a positive outcome occurring to oneself:

You want to apply for a research assistant position. You have 2 weeks to get two letters of reference and to fill out the application form. You needlessly put it off until the last minute and no professor is able to write you a letter in time for the deadline. You apply without the letters, and since no one else applied, the department decides to give you the position.

A sample scenario involving an experienced delay with a negative outcome occurring to oneself:

You are in charge of doing data analysis on SPSS (statistical software) for a presentation. You decide to begin the analysis 2 weeks prior to the date of the presentation in order to be well prepared. You do not have SPSS software on your laptop and you are then forced to go and see your friend in order to use it. You can only work for a few hours a week because your friend has such a busy schedule. At the end of the first week your friend informs you that she accidently deletes the project. You then begin to work on it all over again. The result is that you can’t get it done in time and you do very badly on your presentation.

A sample scenario involving procrastination with a negative outcomes occurring to others:

Michael wants to apply for a research assistant position. He has two weeks to get two letters of reference and to fill out the application form. He needlessly puts it off until the last minute and no professor is able to write him a letter in time for the deadline. The department says that they will not accept his application without letters of reference; therefore he does not get the position.

After reading each of the two scenarios in a given experimental condition (both having the same three-factor combination), participants responded to three self-report items on a 6-point rating scale (1 = *strongly disagree*, 6 = *strongly agree*). The first measure after each scenario consisted of a manipulation check concerning the extent to which the agent was perceived as engaging in procrastination. The second and third measures evaluated to what extent participants perceived the agent to be *morally responsible* for the outcome described as well as *blameworthy* for the outcome. At the end of the study, participants were presented with debriefing information concerning the purpose of the study and dismissed.

## Results

### Preliminary Analyses

Concerning the ANCOVA assumptions, students were randomly assigned to experimental conditions, satisfying the assumption of independence of observations. Next, there was a loss of 29 participants due to missing responses on the study measures. One significant univariate outlier was identified and removed (for the variable “age” using *z*-scores with a criterion of *p* < 0.001, two-tailed), resulting in the final sample of 210 students. With respect to normality, the variables of moral responsibility, blameworthiness, age, and gender were not normally distributed, as assessed by Shapiro–Wilk’s test (*p* < 0.05). Finally, although Levene’s test of homogeneity of variance was significant (responsibility; *p* = 0.015, blameworthiness; *p* < 0.001), our experimental conditions had relatively equal numbers of students, mitigating potential confounds due to high variance combined with proportionally low sample sizes. Homogeneity of regression slopes as interaction terms were not significant for age or gender.

All participants read multiple vignettes, one after the other, in the same order, each reflecting the same combination of three experimental factors. Accordingly, analyses of change over time were not conducted due to possible order effects. Responses for each pair of items following the two scenarios were averaged to create three measures assessing perceived procrastination as a manipulation check [inter-item *r*(211) = 0.78, *p* < 0.001; *M* = 3.79, *SD* = 1.97] as well as moral responsibility [inter-item *r*(211) = 0.55, *p* < 0.001; *M* = 4.19, *SD* = 1.51] and blameworthiness [inter-item *r*(211) = 0.62, *p* < 0.001; *M* = 4.15, *SD* = 1.58] as dependent measures. In addition, the extent to which the experimental variable of primary interest (procrastination versus delay) was uniquely perceived by participants as reflecting procrastination behavior, was further evaluated using an independent samples *t*-test. Results indicated that participants were indeed able to clearly differentiate procrastination from delay scenarios, *t*(209) = 24.21, *p* < 0.001, *d* = 3.33, supporting the efficacy of our main experimental manipulation. Finally, despite a significant correlation between moral responsibility and blameworthiness, *r*(211) = 0.77, *p* < 0.001, both dependent measures were retained in subsequent analyses to evaluate a socio-cognitive as well as more affective variant of perceived intentionality, respectively.

ANCOVA analyses were conducted to evaluate the effects of the experimental conditions as independent variables of Behavior (procrastination versus delay), Outcome (negative versus positive), and Target (self versus other) on moral responsibility and blameworthiness as dependent measures. Age and gender were included as covariates based on prior research showing older individuals to procrastinate less often (e.g., Baumeister et al., 1994; O’Donoghue and Rabin, 1999) and males to be more likely to procrastinate than females (e.g., Milgram et al., 1995; Pychyl et al., 2002; Steel, 2007). In addition, correlations and χ^2^ analyses were conducted to rule out initial differences between our background variables (age and gender), the experimental conditions, and the study measures. Although the results showed no significant differences with respect to the background variables, age and gender were nonetheless included as covariates in the study analyses to maintain consistency with published research and provide a suitably conservative test of the study hypotheses with respect to previously demonstrated potential confounds.

### Main Analysis

**FIGURE 1:**
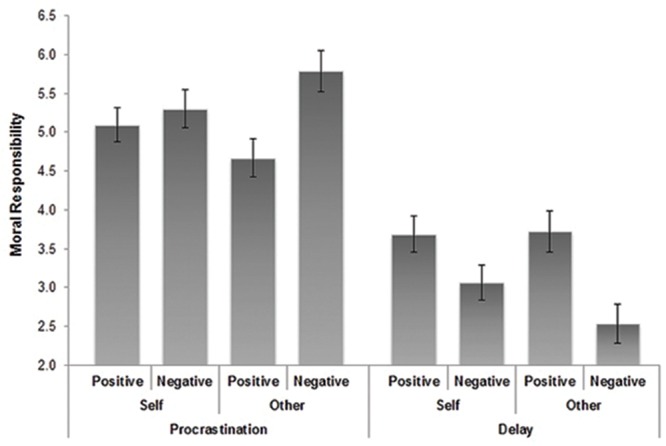
**Three-way interaction between Behavior, Outcome, and Target**.

As expected, a main effect was found for Behavior on blameworthiness showing procrastination (*M* = 5.40, *SD* = 0.78) to be perceived as more blameworthy than delays (*M* = 3.00, *SD* = 1.09), *F*(1,152) = 272.70, *p* < 0.001, $\eta_{p}^{2}$ = 0.64. Additionally, a significant interaction between Behavior and Outcome on blameworthiness was observed, *F*(1,152) = 4.00, *p* = 0.05, $\eta_{p}^{2}$ = 0.03, suggesting that procrastination was seen as more blameworthy when the outcome was negative. Similarly, a main effect of Behavior was found on moral responsibility, *F*(1,152) = 132.36, *p* < 0.001, $\eta_{p}^{2}$ = 0.47, with results showing procrastination (*M* = 5.17, *SD* = 1.10) to be viewed as involving more moral responsibility than delays (*M* = 3.23, *SD* = 1.20). Although a significant two-way interaction between Behavior and Outcome was observed, *F*(1,152) = 21.43, *p* < 0.001, $\eta_{p}^{2}$ = 0.12, this effect was qualified by a three-way interaction effect, *F*(1,152) = 4.02, *p* = 0.05, $\eta_{p}^{2}$ = 0.03. As presented in **Figure 1**, participants tended to attribute greater moral responsibility to procrastination resulting in negative versus positive outcomes, but only when evaluating other individuals. In contrast, delays were evaluated as involving lower levels of moral responsibility when resulting in negative versus positive outcomes, especially when the delay was experienced by others versus oneself.

To further probe the significant interaction between Target, Behavior, and Outcome, we examined the simple effects of Behavior and Outcome by Target level (self versus other). There was a significant simple interaction effect between Behavior and Outcome for participants in the “other” condition, *F*(1,152) = 19.67, *p* < 0.001, but not for those in the “self” condition, *F*(1,152) = 3.78, *p* = 0.054.

## Discussion

In this study, we investigated how students perceive procrastination, as opposed to experienced delays, through the theoretical lenses of social psychology, experimental philosophy, and educational psychology. Utilizing an experimental, scenario-based approach, our results showed students’ intention-related evaluations of procrastination and delay to be moderated by the outcome (positive versus negative) as well as who was being judged (self versus other). First, significant main effects showed participants to clearly distinguish between procrastination and delay experiences. In support of Hypothesis 1, students evaluated procrastination as more blameworthy and morally responsible as compared to delays.

Second, in accordance with Hypothesis 4, two-way interaction effects revealed that the degree of responsibility attributed to oneself or others depended on whether the outcome of procrastination or delay was positive or negative. Whereas students who engaged in procrastination were generally perceived as higher in moral responsibility when the outcome was negative, delays were instead viewed as *less* deserving of moral responsibility when they resulted in failure. Conversely, positive consequences of procrastination were viewed as involving less moral responsibility, with success following delay experiences viewed as higher in moral responsibility (e.g., giving credit for successful self-regulation). This finding is consistent with Knobe (2003) who suggests that negative outcomes with moral implications tend to be immediately perceived as more intentional in nature. Moreover, this result is in line with attribution theory in showing failure despite effort to imply a lack of ability resulting in lower perceived responsibility and greater sympathy (e.g., Weiner, 2000).

Additionally, an unanticipated three-way interaction was observed showing this pattern of results to be further moderated by whether participants were judging themselves or others. Whereas students generally viewed procrastination as deserving of moral responsibility following failure, procrastination that specifically resulted in failure was viewed as involving more moral responsibility when it happened to *others* as opposed to oneself. More specifically, although students tended to view themselves as morally responsible for procrastination *despite* the outcome, they rated other students less responsible than themselves when procrastination did not impair performance and more harshly than themselves when procrastination had negative consequences. This finding is novel in research on procrastination in showing outcome and target to moderate perceptions of intentionality and expands on social-psychological theories of perception biases in showing individuals to perceive others as more culpable due to dispositional factors following failure, specifically failure due to *procrastination*.

The results from the present study also help explain why research has consistently shown procrastination to negatively correlate with academic performance (Kim and Seo, 2015). According to our results as outlined in **Figure 1**, students rated procrastination that resulted in failure as more immoral when occurring to others than to themselves, thus suggesting that procrastination may negatively impact student performance in part due to a relative lack of perceived responsibility for their study behaviors. This interpretation is consistent with research suggesting that self-forgiveness for misbehavior requires one to first take responsibility for the transgression (Wohl et al., 2010). Finally, information concerning the moral implications of procrastination is vital for developing intervention programs to assist students, specifically concerning efforts to promote adaptive student cognitions (e.g., perceived responsibility) concerning their procrastination behavior.

Moreover, the present findings contribute to research on procrastination in highlighting the social implications of this detrimental behavior in educational settings. Although previous research has convincingly demonstrated the negative personal consequences of procrastination with respect to academics (Schraw et al., 2007) and well-being (Sirois and Pychyl, 2013), it is limited with respect to its social implications. Given that perceptions of blameworthiness and responsibility are clearly linked to tasks that involve social obligation (Eshleman, 2009), it is perhaps not surprising that procrastination was linked to intentionality-related beliefs involving others as evaluated using both a cognitive measure (responsibility) and more affective measure (blame; see Weiner, 2006).

### Limitations and Future Directions

Multiple limitations are to be considered when interpreting the results of the present study. First, whereas the present study followed from recent research on the utility of single-item self-report measures in motivation and blameworthiness research (Inbar et al., 2012; Gogol et al., 2014), future studies employing more intensive qualitative or multi-item measures (Lay, 1986), as well as objective indicators (e.g., observational data) are encouraged. Furthermore, the present study did not take into account the potential influence of other variables such as demographics (ethnicity or socio-economic status), psychosocial variables (personality traits), or contextual factors (years in program, domain). Second, the present study relied on students’ self-report responses to scenario measures warranting further research to evaluate responses in real time (e.g., experience sampling methods; Nett et al., 2012) to naturally occurring or manipulated behavior of an actual target individual. Third, it is important to note that the main effect for Outcome experimental condition (positive versus negative) on blameworthiness, as well as for the 3-way interaction results, were small in magnitude (effect size, Cohen, 1988; for a critique, see Cortina and Landis, 2009). Fourth, although age and gender were controlled for in the analysis, the demographic composition of the sample (e.g., 75% female, university students) warrants further research to examine whether our findings generalize across gender and to populations in other achievement contexts (e.g., employment settings). Nevertheless, these preliminary empirical findings are encouraging in suggesting that moral perceptions of procrastination and its outcomes do differ in educational settings depending on whether or not it is occurring to oneself vs. others, underscoring the importance of further exploring social perceptions of procrastination in other domains (e.g., employment) utilizing more intensive self-report as well as objective measures.

## Author Contributions

SR conducted data collection, statistical analysis, and manuscript writing. NH and TP contributed to manuscript writing.

## Conflict of Interest Statement

The authors declare that the research was conducted in the absence of any commercial or financial relationships that could be construed as a potential conflict of interest.
